# Development and trends in research on hypertension and atrial fibrillation: A bibliometric analysis from 2003 to 2022

**DOI:** 10.1097/MD.0000000000038264

**Published:** 2024-05-24

**Authors:** Nan Tang, Qiang Zhou, Shuang Liu, Kangming Li, Zhen Liu, Qingdui Zhang, Huamei Sun, Cheng Peng, Ji Hao, Chunmei Qi

**Affiliations:** a Department of Cardiology, The Second Affiliated Hospital of Xuzhou Medical University, Xuzhou, China.

**Keywords:** atrial fibrillation, bibliometric analysis, cardiovascular diseases, hypertension, research trends

## Abstract

**Background:**

This study aimed to comprehensively analyze research related to hypertension and atrial fibrillation, 2 common cardiovascular diseases with significant global public health implications, using bibliometric methods from 2003 to 2022.

**Methods:**

From the Web of Science Core Collection database, literature on the theme of hypertension and atrial fibrillation was retrieved. Subsequently, comprehensive bibliometric analyses were conducted across multiple dimensions utilizing software tools such as VOSviewer, Citespace, Pajek, Scimago Graphica, and ClusterProfiler. These analyses encompassed examinations of the literature according to country/region, institution, authors, journals, citation relationships, and keywords.

**Results:**

It revealed an increasing interest and shifting focus in research over the years. The analysis covered 7936 relevant publications, demonstrating a gradual rise in research activity regarding hypertension combined with atrial fibrillation over the past 2 decades, with a stable growth trend in research outcomes. Geographically, Europe and the Americas, particularly the United States, have shown the most active research in this field, while China has also gained importance in recent years. Regarding institutional contributions, internationally renowned institutions such as the University of Birmingham and the Mayo Clinic have emerged as core forces in this research direction. Additionally, Professor Lip Gregory, with his prolific research output, has stood out among numerous scholars. The American Journal of Cardiology has become a primary platform for publishing research related to hypertension and atrial fibrillation, highlighting its central role in advancing knowledge dissemination in this field. The research focus has shifted from exploring the pathophysiological mechanisms to investigating the treatment of complications and risk factors associated with hypertension and atrial fibrillation. Future research will focus on in-depth exploration of genetic and molecular mechanisms, causal relationship exploration through Mendelian randomization studies, and the application of machine learning techniques in prediction and treatment, aiming to promote the development of precision medicine for cardiovascular diseases.

**Conclusion:**

In conclusion, this study provides a comprehensive overview of the developmental trajectory of research on hypertension and atrial fibrillation, presenting novel insights into trends and future research directions, thus offering information support and guidance for research in this crucial field of cardiovascular medicine.

## 1. Introduction

As global health issues become increasingly prominent, cardiovascular diseases have emerged as the leading public health challenge worldwide. Among them, hypertension and atrial fibrillation, as 2 common and serious chronic health conditions, have attracted widespread attention in recent years due to their interrelationship and independent effects in research.^[1]^ Hypertension, as one of the most common non-communicable diseases globally, affected approximately 1.2 billion people worldwide in 2019, according to the World Health Organization, and this number is projected to rise to 1.5 billion by 2030.^[2]^ As a major risk factor, hypertension is associated with chronic conditions such as heart disease, stroke, and kidney disease, leading to approximately 10 million deaths annually.^[3,4]^ Hypertension not only directly causes structural and functional changes in the heart and vascular system but is also closely linked to various other cardiovascular complications. Meanwhile, atrial fibrillation is the most common arrhythmia encountered in clinical practice, with its incidence significantly increasing with age and being particularly prominent among patients with hypertension. Currently, approximately 33 million people globally are estimated to have atrial fibrillation, with a 31% increase in incidence over the past 2 decades, and further increases are anticipated.^[5,6]^ Atrial fibrillation significantly increases the risk of complications such as stroke, heart failure, and cognitive impairment, severely affecting patients’ quality of life and prognosis.^[7]^ From a pathophysiological perspective, there exists a close intrinsic connection between hypertension and atrial fibrillation.^[8]^ Hypertension exacerbates atrial electrophysiological abnormalities by promoting left atrial remodeling, inflammatory responses, oxidative stress, and autonomic nervous system imbalance, thereby increasing the risk of atrial fibrillation occurrence.^[9]^ At the same time, atrial fibrillation patients, due to hemodynamic disturbances, further increase the likelihood of thromboembolic events such as stroke in hypertensive patients.^[10]^ Therefore, prevention, diagnosis, and management of these intertwined cardiovascular diseases are crucial for enhancing public health levels.

Given the high comorbidity rates of these 2 diseases and their significant impact on health outcomes, research on hypertension and atrial fibrillation in recent years has exhibited multidimensional, interdisciplinary characteristics, covering various aspects including epidemiology, exploration of basic mechanisms, improvement of diagnostic methods, optimization of prevention strategies, and innovation in treatment methods. This is attributable to advancements in scientific methodology, technological innovation, and the accumulation of medical insights. Epidemiological studies have revealed the distribution patterns, determinants, and burden characteristics of hypertension and atrial fibrillation globally.^[11–13]^ Basic research has delved into the pathological mechanisms, molecular markers, and genetic basis of these diseases.^[14–16]^ Clinical research has explored diagnostic methods, therapeutic interventions, and prognostic markers associated with hypertension and atrial fibrillation.^[17–19]^ Meanwhile, translational research has led to the development of prevention strategies, treatment guidelines, and patient education initiatives.^[20–22]^ Despite the progress made, the fields of hypertension and atrial fibrillation research still face numerous challenges and gaps, including incomplete understanding of the pathophysiological mechanisms, inadequate early detection and intervention, urgent need to improve treatment efficacy and safety, and the demand for strengthened preventive measures.^[23–25]^ Therefore, conducting a comprehensive bibliometric analysis is crucial for elucidating the current status, identifying emerging trends, and predicting future research directions in the fields of hypertension and atrial fibrillation.

Bibliometrics is a discipline that utilizes mathematical, statistical, and other methods to quantitatively analyze scientific literature.^[26]^ This analysis can reveal the quantity, structure, and evolutionary characteristics of scientific literature, thereby uncovering the development patterns and knowledge structures of scientific research.^[27]^ In recent years, the explosive growth of scientific literature data and continuous improvement of bibliometric software tools have facilitated the widespread application of bibliometric methods across various disciplines.^[28,29]^ In this context, this study aims to apply bibliometric methods to systematically review and quantitatively analyze relevant academic literature, exploring the current status, emerging themes, and development trends in the research fields of hypertension and atrial fibrillation. This study primarily searched the Web of Science database for literature related to hypertension and atrial fibrillation from 2003 to 2022, yielding a total of 7936 valid articles. Bibliometric analysis involved the use of various software tools such as VOSviewer, Citespace, Pajek, Scimago Graphica, and R package Clusterprofiler. Through multidimensional studies on geographic distribution, institutional affiliation, author patterns, journal preferences, citation dynamics, and thematic keyword clustering, this study aims to reveal current trends, define thematic clusters, and uncover emerging research frontiers. This endeavor aims to provide valuable reference points for researchers and support information for scientific research planning and decision-making, thereby promoting further development in the fields of hypertension and atrial fibrillation research.

## 2. Data sources and analysis methods

### 2.1. Data source

**Figure 1.**
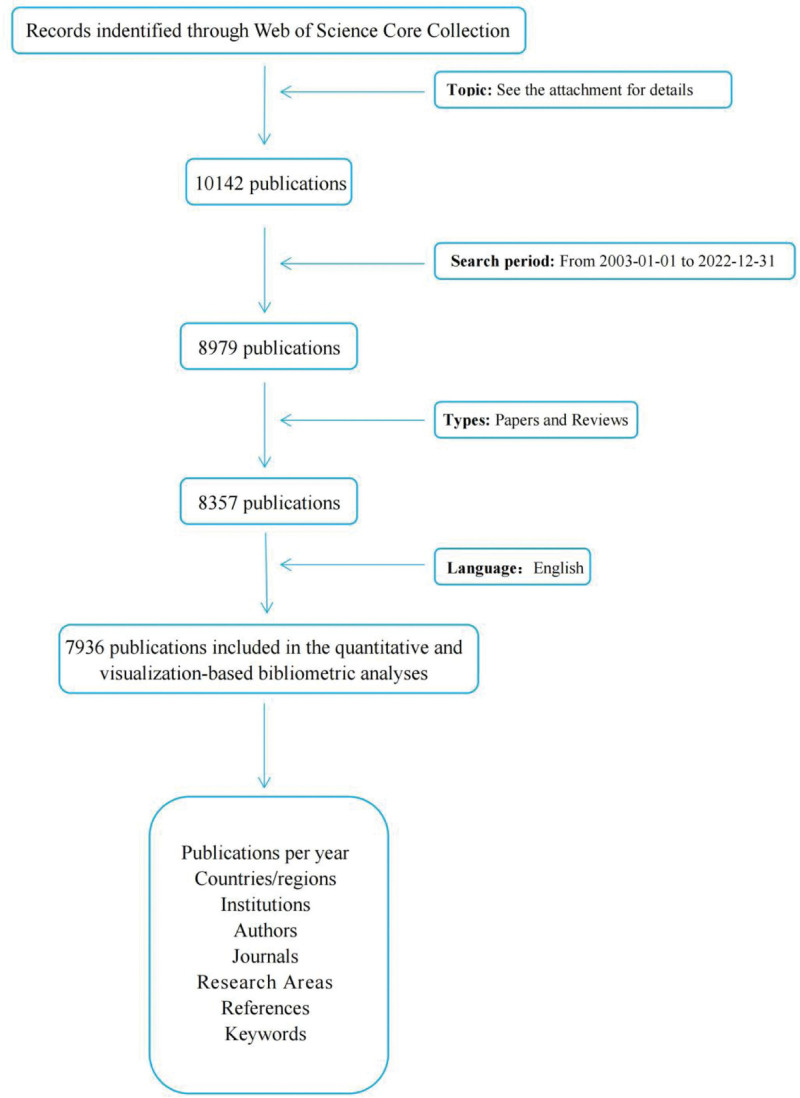
Retrieval flowchart.

Web of Science database: The Web of Science Core Collection database was searched using the query title and subject (TS)=(“atrial fibrillation” OR “auricular fibrillation” OR “persistent atrial fibrillation” OR “atrium fibrillation” OR “paroxysmal atrial fibrillation”) AND TS = (hypertension), with the search time limited from 2003-01-01 to 2022-12-31. The inclusion criteria were papers and reviews related to the search, and letters, brief reports, book reviews, etc were excluded, resulting in 7936 articles. The full records and cited references of the search results were exported in plain text format. The data were used for the visualization analysis of country/region, institution, author, journal, research field, co-cited literature, keyword, etc. The specific search process is shown in Figure 1, and the search was performed within 1 day (August 25, 2023) to avoid the bias caused by the daily database update. The data obtained from the search were secondary data, which did not contain any personal information, and no informed consent was required.

### 2.2. Analysis method

Using bibliometric methods, the retrieved literature was analyzed by VOSviewer 1.6.18 (Centre for Science and Technology Studies, Leiden University, The Netherlands), Citespace 6.1.6 (Chaomei Chen, China), Pajek 64 5.16 (University of Ljubljana, Slovenia), Scimago Graphica 1.0.35 (https://www.graphica.app/, United States), and R package Clusterprofiler software for country, institution, author, journal, research field, co-cited literature, and keyword visualization analysis, and related visualization maps were drawn to analyze the research status, hotspots, and trends of this study.

VOSviewer 1.6.18 (Centre for Science and Technology Studies, Leiden University, The Netherlands) and Pajek 64 5.16 (University of Ljubljana, Slovenia) were used to co-occur analysis of country, institution, author, journal publication, and research field. In the analysis of country, institution, author, journal publication, and research field, different colors represent different clusters. In the graph drawn by the software, a circle and a text label represent a node, and the size of the node is represented by the size of the circle; the connection between nodes represents the co-occurrence relationship; the thickness of the connecting line segment represents the co-occurrence strength.

By employing Citespace 6.1.6 (Chaomei Chen, China) software, we utilized data visualization techniques to analyze journals, co-cited literature, and keywords, thereby constructing visualization maps. Furthermore, we generated graphical representations showcasing the top 10 keywords based on their emergence strength. In the journal overlay map, the left side displays citing journals, while the right side exhibits cited journals. Each point in the graph symbolizes a journal, and the curves connecting the 2 halves illustrate citation links, offering a holistic understanding of interdisciplinary relationships within this domain. The co-cited literature map was created using specific parameters in Citespace: time range (2003–2022), yearly slicing (1), and selection criterion (k = 1). Distinct circles featured in the co-cited literature map correspond to different co-cited references, with the size of each circle denoting the number of citations it has received. The connections between circles represent co-citation relationships, while the colors of the circles, connections, and modules indicate diverse clusters. Additionally, the size and color of the annual rings encompassed within each circle convey the quantity and corresponding time period of the cited literature.

R package Clusterprofiler was used to analyze the cooperation of countries.

Scimago Graphica 1.0.35 (https://www.graphica.app/, United States) software was used to analyze the trend of keyword popularity over time, with the total year length (2003–2022), the year per time period (4 years), and the selection criterion (the top 50 keywords in each slice year). The connection between keywords in each time period represents the trend of keyword popularity in different time periods. Hollow circles are the time periods when the keyword first entered the top 50 in 2003–2022, and solid circles represent the end time period when the keyword appeared in the top 50.

## 3. Results

### 3.1. Publication trend analysis

**Figure 2.**
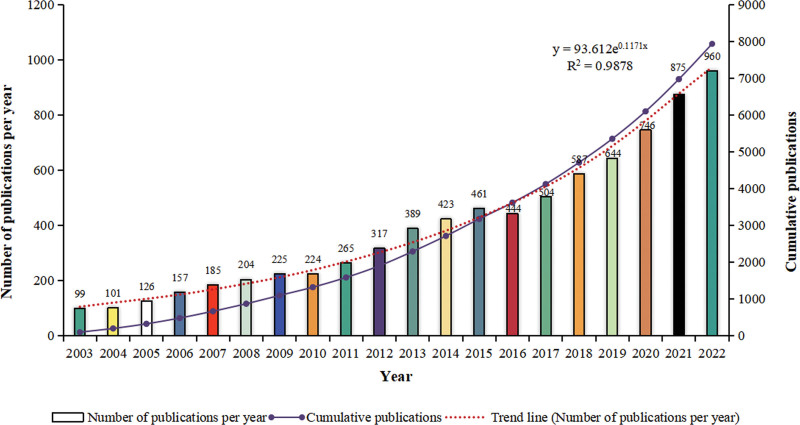
Annual and cumulative publication trend analysis.

According to the inclusion criteria, a total of 7936 articles related to hypertension and atrial fibrillation research field were retrieved from the Web of Science core collection. The annual publication volume of the related field is shown in Figure 2. The publication volume and cumulative publication volume of the related research literature showed an increasing trend year by year. 2015 and 2022 were the peak periods of publication, with more research results on hypertension and atrial fibrillation produced in these 2 years, and the publication volume was more concentrated, in the hot spot period of research. According to the publication trend line, an exponential function of the annual publication trend was created: y = 93.612e^0.1171x^ (R^2^ = 0.9878, x = year-2002, y is the annual publication volume), with a good fit, and the publication volume in 2023 was predicted to exceed 1000. It can be seen that the research on hypertension and atrial fibrillation-related field is increasingly valued by scholars, and the research interest will continue.

### 3.2. Country and institution co-occurrence analysis

**Figure 3.**
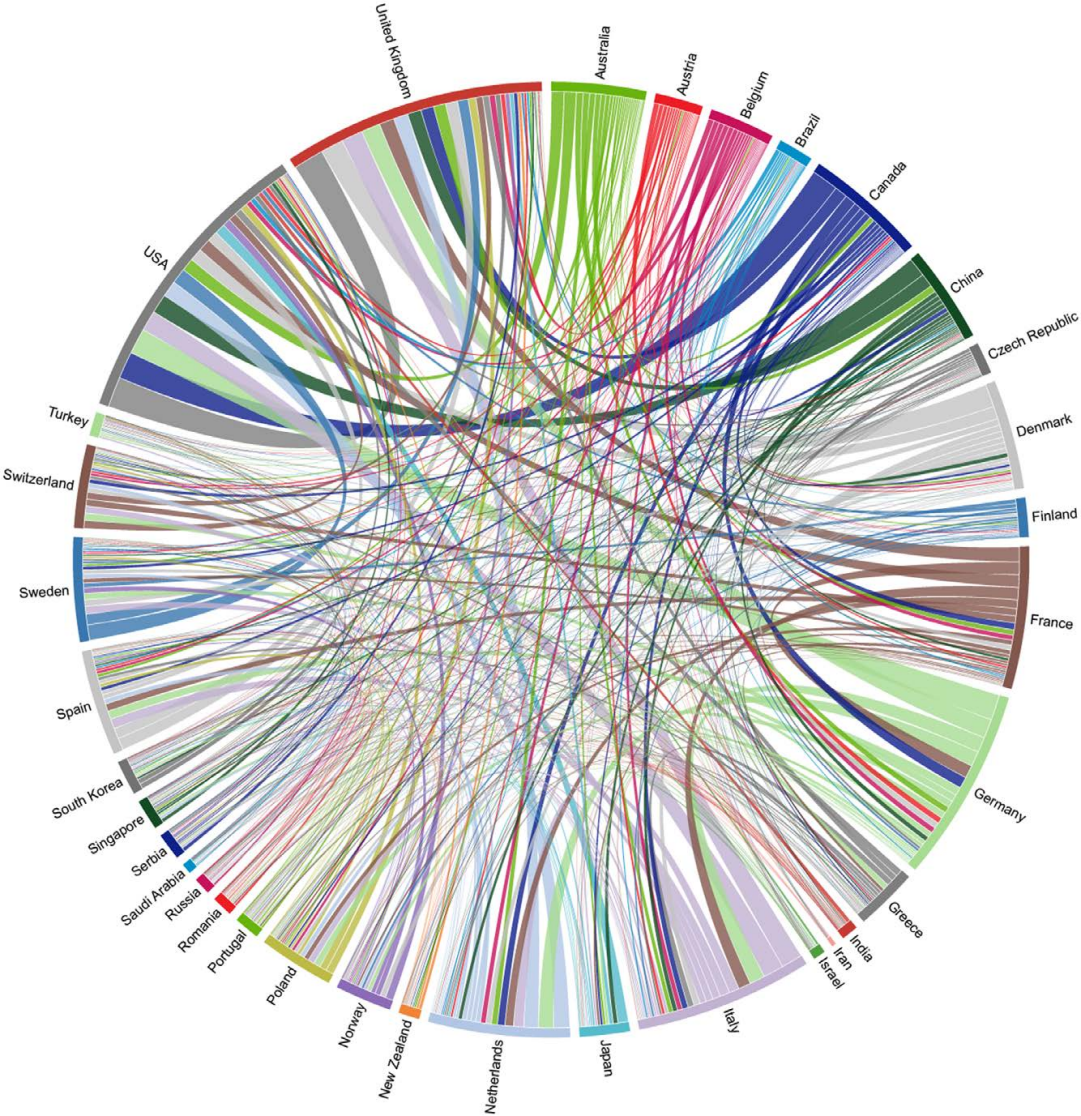
Country relationship analysis map.

**Figure 4.**
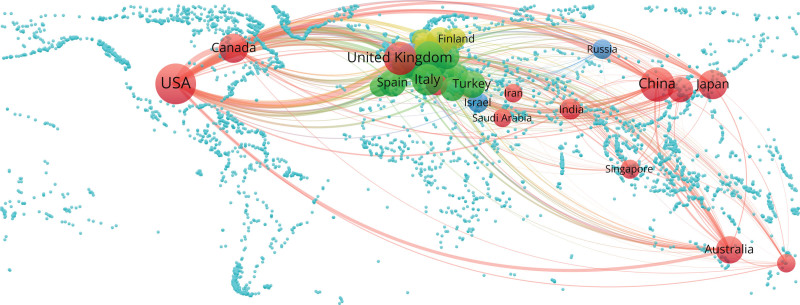
Country co-occurrence map.

By analyzing the countries and institutions, we can understand the global distribution of the research field related to hypertension and atrial fibrillation, and provide important information for understanding the global research status of this field. The results show that a total of 125 countries and 8821 institutions published 7936 articles on hypertension and atrial fibrillation-related research. Combining Figure 3, Figure 4, and Table 1, we can see that: the United States is the leader and pioneer of this field, with a publication volume of 2520 articles, far exceeding the second-ranked China 1127 articles. It is also the earliest country to conduct research in this field, and it has the strongest willingness to cooperate with other countries, especially with the United Kingdom (UK). Developed countries such as Europe and America are the main research forces in this field, and their publication volume and cooperation degree are relatively high. In addition to the United States and China, the top 5 countries in terms of publication volume are the UK (907 articles), Italy (691 articles), and Germany (583 articles). The main researchers from each country are as follows: Soliman Elsayed, Chen Shih-Ann, Lip Gregory, Maggioni Aldo, Boehm Michael. These individuals are key contributors in their respective countries within the research field.

**Table 1:** Top 5 countries by publication volume.

Rank	Country	Publications	Citations	Average citations	Main researchers
1	United States	2520	147934	59	Soliman Elsayed
2	China	1127	24823	22	Chen Shih-Ann
3	United Kingdom	907	61431	68	Lip Gregory
4	Italy	691	35424	51	Maggioni Aldo
5	Germany	583	34887	60	Boehm Michael

**Figure 5.**
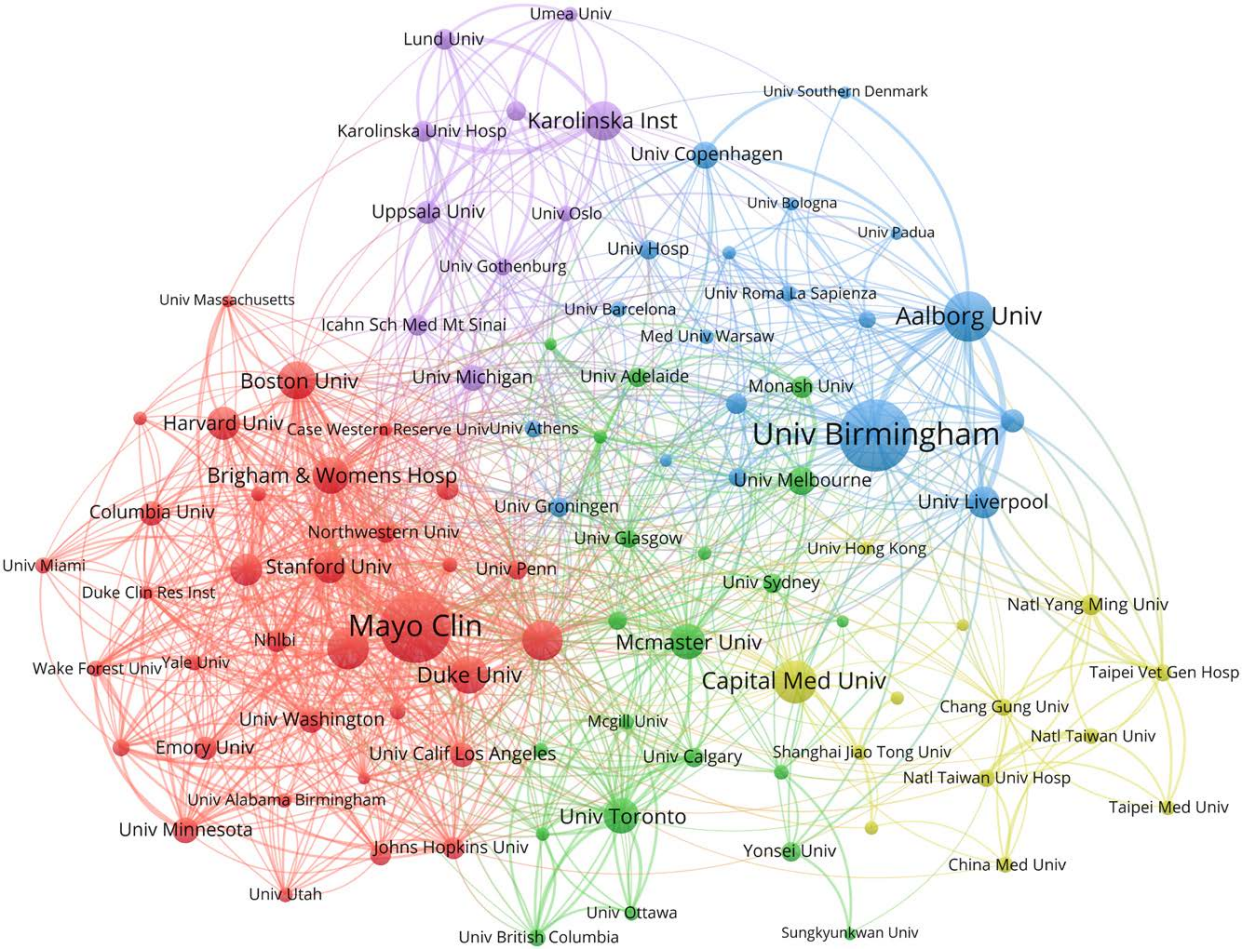
Institution co-occurrence map.

As shown in Figure 5 and Table 2, Aalborg University is the most active institution in this field, with the strongest willingness to cooperate with other institutions, and the closest cooperation with University of Birmingham. The top 5 institutions in terms of publication volume are University of Birmingham (223 articles), Mayo Clinic (219 articles), Capital Medical University (130 articles), and Massachusetts General Hospital (125 articles). These institutions are important and influential institutions in this field, mostly concentrated in Europe and America, mainly universities and medical centers, and they have close cooperation with each other, forming a cross-regional international cooperation network.

**Table 2:** Top 5 institutions by publication volume.

Rank	Institution	Publications	Citations	Average citations
1	University of Birmingham	223	23184	104
2	Mayo Clinic	219	18591	85
3	Aalborg Universitet	152	5677	37
4	Capital Medical University	130	2532	19
5	Massachusetts General Hospital	125	17062	136

### 3.3. Author co-occurrence analysis

**Figure 6.**
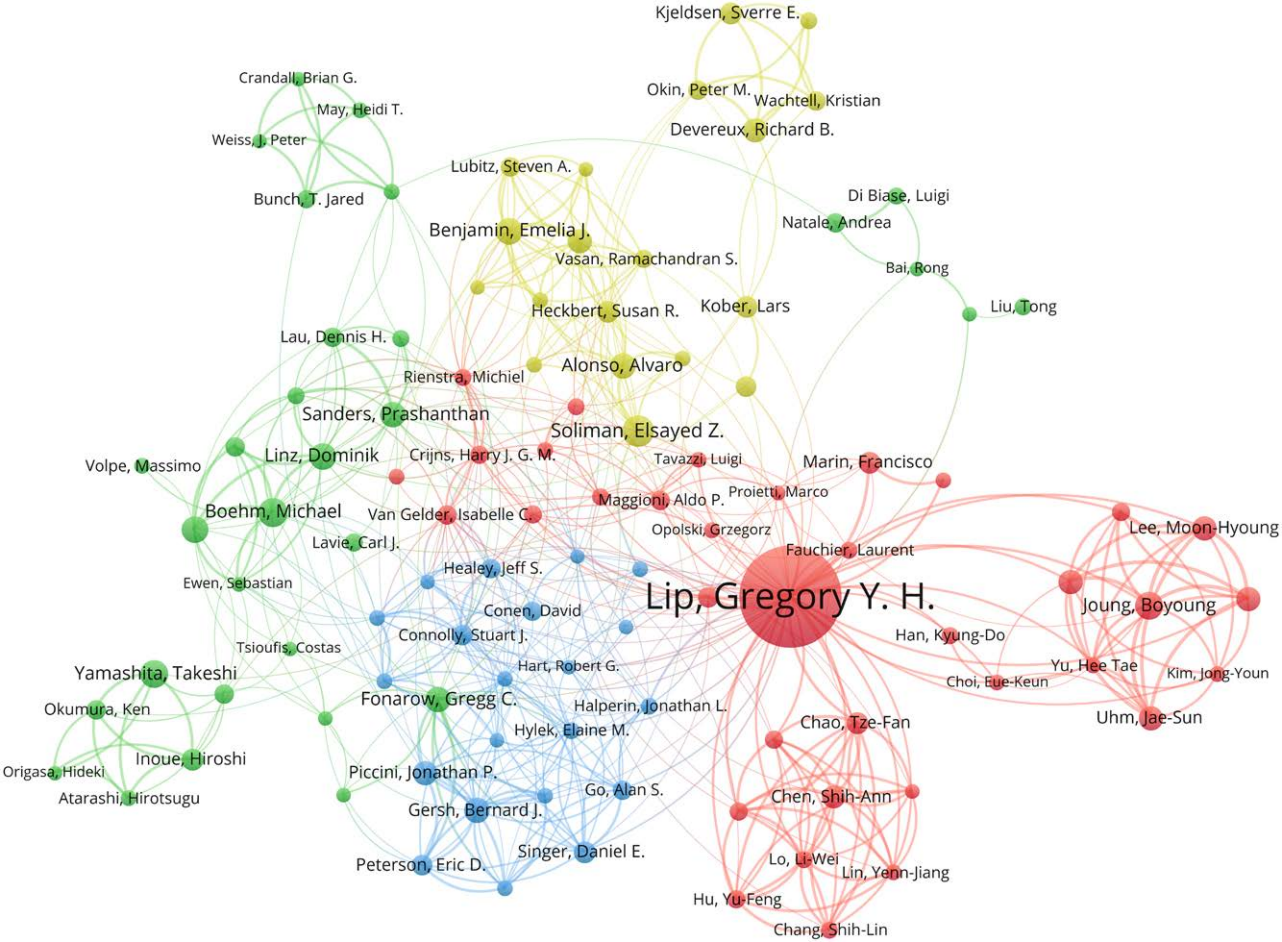
Author co-occurrence map.

The author scientific collaboration network visualization map helps to provide information on influential research teams and potential collaborators, thus providing reference for researchers to carry out cooperation. The results showed that 40011 authors published 7936 papers on hypertension and atrial fibrillation-related research. Figure 6 shows the collaboration visualization map of these authors, where different colors of dots represent different authors, and the connection between dots represents the cooperation relationship, and the thicker the connection, the more cooperation times. From the figure, it can be seen that Lip Gregory is the most active author in this field, with the strongest willingness to cooperate with other authors, and Joung Boyoung and Uh Jae-Sun are the closest partners, while the cooperation between other authors or teams is relatively less, indicating that the cooperation network in this field needs to be expanded and strengthened. Table 3 lists the top 5 authors in terms of publication volume in this field, who are the high-yield authors in this field and have made important contributions to the research of this field. Among them, Lip Gregory has the highest publication volume, reaching 248 papers, and he also participated in the formulation of several guidelines, laying an important foundation for the research of this field.

**Table 3:** Top 5 authors by publication volume.

Rank	Author	Publications	Citations	Average citations
1	Lip Gregory	248	24005	97
2	Soliman Elsayed	45	2042	45
3	Boehm Michael	40	1686	42
4	Joung Boyoung	39	1038	27
5	Yamashita Takeshi	38	1092	29

### 3.4. Journal co-occurrence analysis

**Figure 7.**
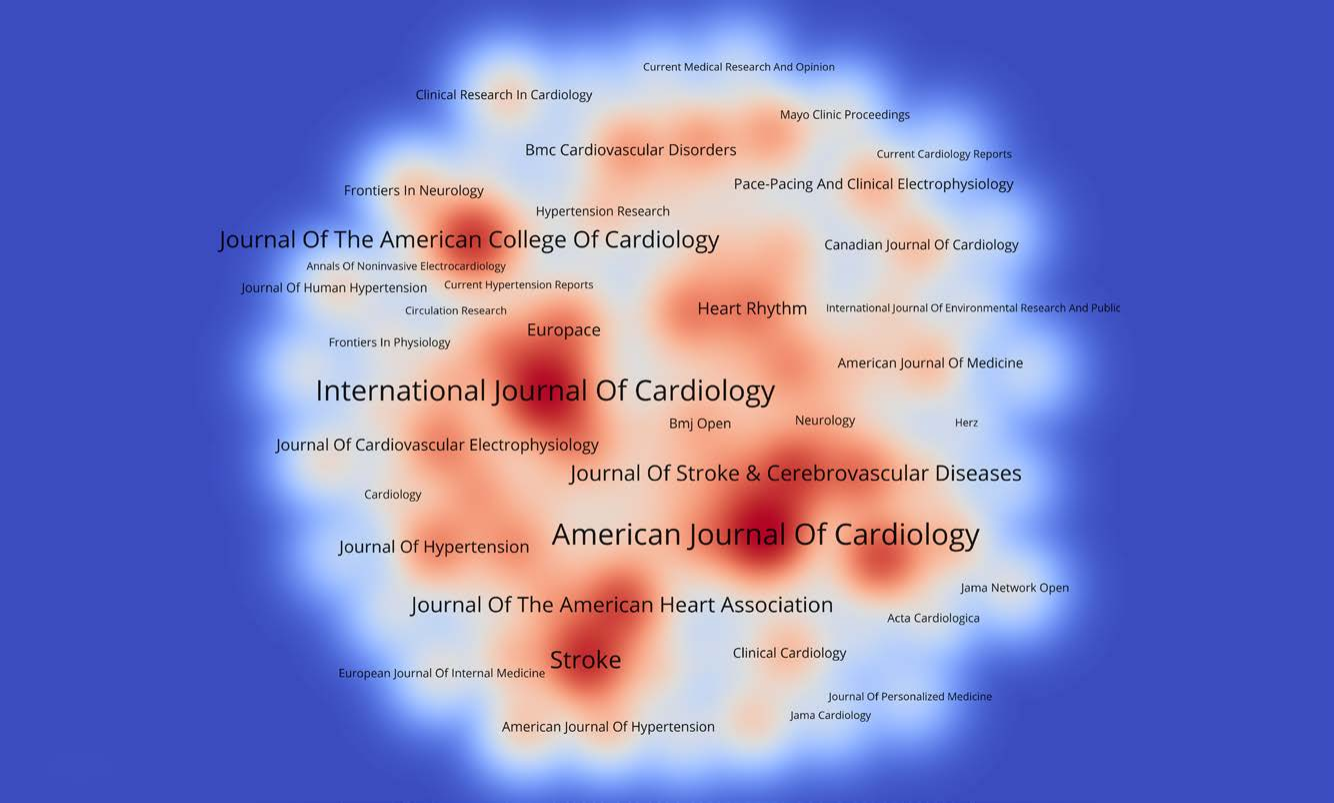
Journal publication heat map.

By analyzing the distribution of core journals in the research field related to hypertension and atrial fibrillation, important journals in the research field can be found, which provide important support for finding high-quality literature. The results showed that 1405 journals published 7936 articles on hypertension and atrial fibrillation-related research. Figure 7 shows the publication heat map of these journals, where the darker the color, the higher the publication volume. Among them, American Journal of Cardiology journal had the highest publication volume, reaching 212 articles, followed by International Journal of Cardiology and Stroke, with 195 and 153 articles respectively. These journals are the main publication platforms for articles in this field.

**Figure 8.**
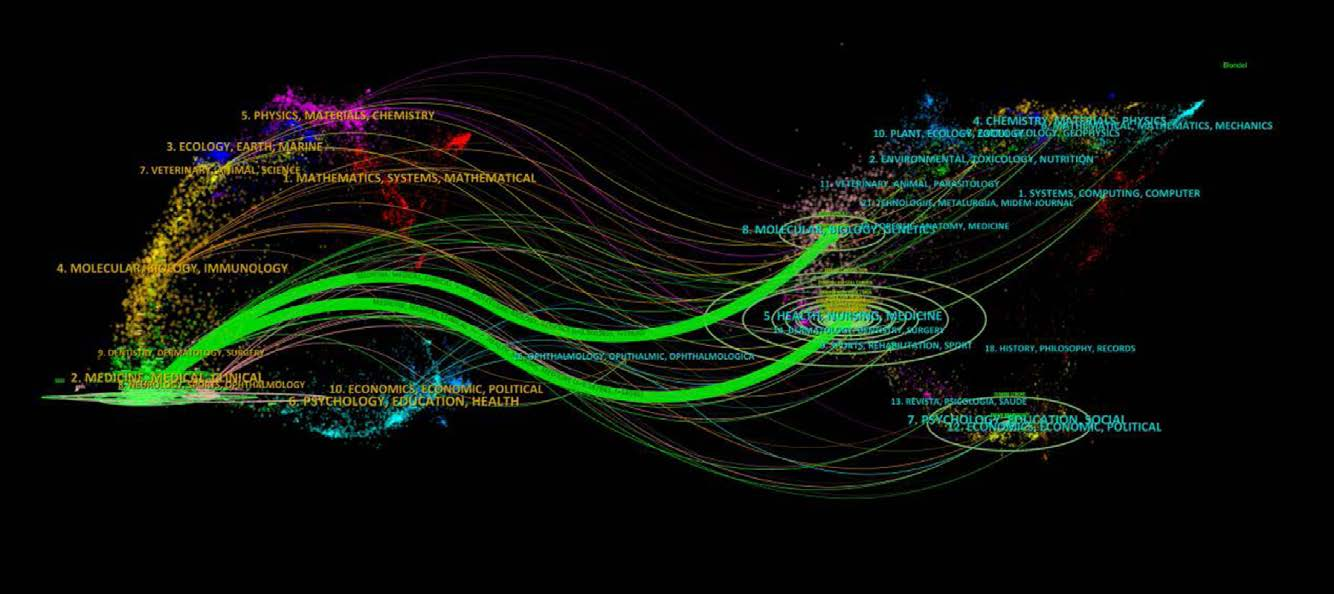
Journal overlay map.

Figure 8 presents a double overlay analysis map of journals, illustrating the relationship between hypertension and atrial fibrillation research as well as the main research disciplines. The map is divided into 2 sections: the left side represents the citing journals, while the right side represents the cited journals. The figure highlights that publications on hypertension and atrial fibrillation research are predominantly found in journals within the fields of Medicine, Medical, and Clinical. On the other hand, citations are primarily concentrated in journals related to Molecular Biology, Genetics, Health, Nursing, and Medicine. Each point on the map represents a journal, and the curves between the points indicate the citation relationships, with thicker curves representing higher citation frequencies. These curves unveil the interdisciplinary nature of this field and demonstrate the connections between different journals.

### 3.5. Research field co-occurrence analysis

**Figure 9.**
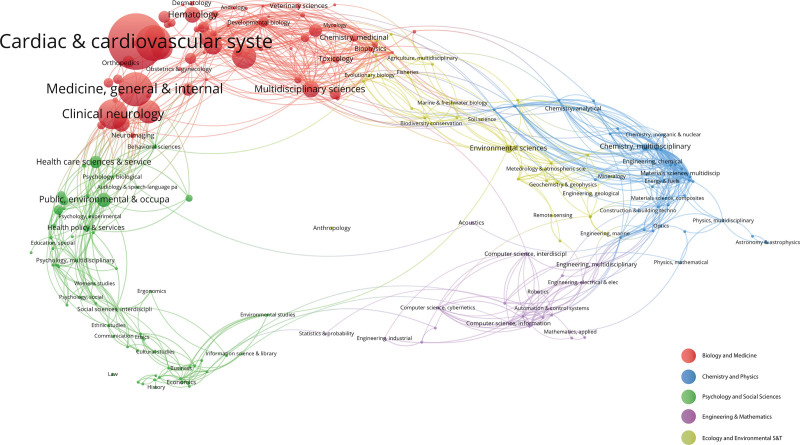
Research field co-occurrence map.

The 227 literature field categories retrieved from the Web of Science core collection database were statistically analyzed and visualized by VOSviewer software, and the 7936 articles on hypertension and atrial fibrillation-related research were clustered into 5 major fields. As shown in Figure 9, different colors of circles represent different field clusters, and it can be seen that hypertension and atrial fibrillation-related research mainly focuses on the “Biology and Medicine” field, among which the subfield of “Cardiac & cardiovascular systems” has a higher proportion.

### 3.6. Literature co-citation analysis

**Figure 10.**
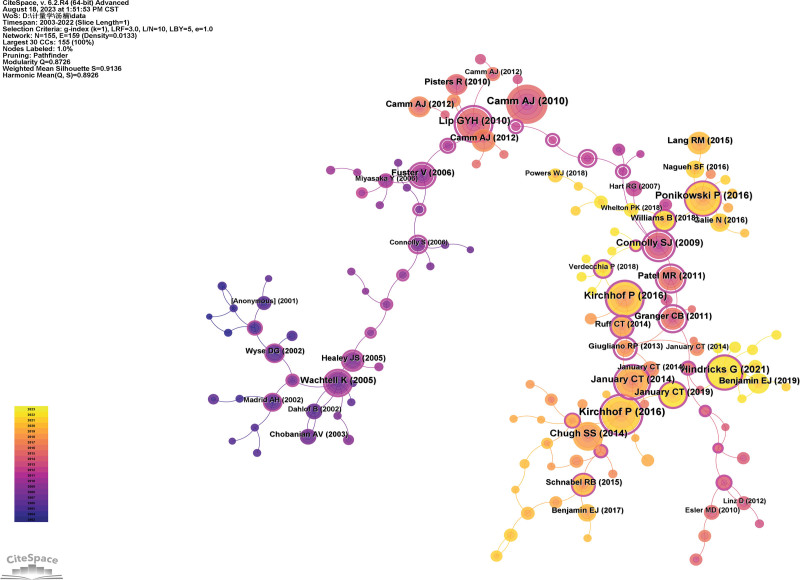
Co-cited literature visualization map.

By mining co-cited literature in a particular field, one can efficiently and effectively pinpoint the foundational knowledge of the discipline and obtain highly influential references within the field through quantitative analysis of co-citation frequency and centrality. The co-citation visualization map generated by Citespace 6.1.6 is shown in Figure 10. Table 4 lists the top 10 co-cited documents sorted by co-citation frequency. Combined with chart analysis, it is evident that the top 10 cited articles mainly involve guidelines for risk management of atrial fibrillation, comparative studies on the efficacy and bleeding risk of anticoagulant drugs in patients with atrial fibrillation, and stroke prevention research. The most frequently co-cited article is the “Guidelines for the Management of Atrial Fibrillation: the Task Force for the Management of Atrial Fibrillation of the European Society of Cardiology” published in 2010.^[30]^ The article with the highest centrality is “Refining clinical risk stratification for predicting stroke and thromboembolism in atrial fibrillation using a novel risk factor-based approach: the euro heart survey on atrial fibrillation”^[31]^ with a centrality of 0.95, indicating its significant impact in the field and its status as a core classic document in the management of atrial fibrillation. Among the top 10 highly cited documents, 4 were published in the European Heart Journal.

**Table 4:** Top 5 literature by co-citation frequency.

Rank	Frequency	Literature	Centrality	Author (yr)	Journal
1	241	Guidelines for the management of atrial fibrillation: the Task Force for the Management of Atrial Fibrillation of the European Society of Cardiology	0.02	Camm AJ (2010)	European Heart Journal
2	228	2016 ESC Guidelines for the management of atrial fibrillation developed in collaboration with EACTS	0.73	Kirchhof P (2016)	Europace
3	183	2016 ESC Guidelines for the diagnosis and treatment of acute and chronic heart failure: The Task Force for the diagnosis and treatment of acute and chronic heart failure of the European Society of Cardiology Developed with the special contribution of the Heart Failure Association of the ESC	0.19	Ponikowski P (2016)	European Heart Journal
4	176	2016 ESC Guidelines for the management of atrial fibrillation developed in collaboration with EACTS	0.49	Kirchhof P (2016)	European Heart Journal
5	167	Corrigendum to: 2020 ESC Guidelines for the diagnosis and management of atrial fibrillation developed in collaboration with the European Association of Cardio-Thoracic Surgery (EACTS)	0.22	Hindricks G (2021)	European Heart Journal
6	165	Refining clinical risk stratification for predicting stroke and thromboembolism in atrial fibrillation using a novel risk factor-based approach: the euro heart survey on atrial fibrillation	0.95	Lip GYH (2010)	Chest
7	143	2014 AHA/ACC/HRS guideline for the management of patients with atrial fibrillation: a report of the American College of Cardiology/American Heart Association Task Force on Practice Guidelines and the Heart Rhythm Society	0.70	January CT (2014)	Journal of the American College of Cardiology
8	139	Worldwide epidemiology of atrial fibrillation: a Global Burden of Disease 2010 Study	0.02	Chugh SS (2014)	Circulation
9	118	Dabigatran vs warfarin in patients with atrial fibrillation	0.92	Connolly SJ (2009)	The New England Journal of Medicine
10	99	Rivaroxaban vs warfarin in nonvalvular atrial fibrillation	0.90	Patel MR (2011)	The New England Journal of Medicine

ACC = American College of Cardiology, AHA = American Heart Association, ESC = European Society of Cardiology.

### 3.7. High-frequency keyword analysis

**Figure 11.**
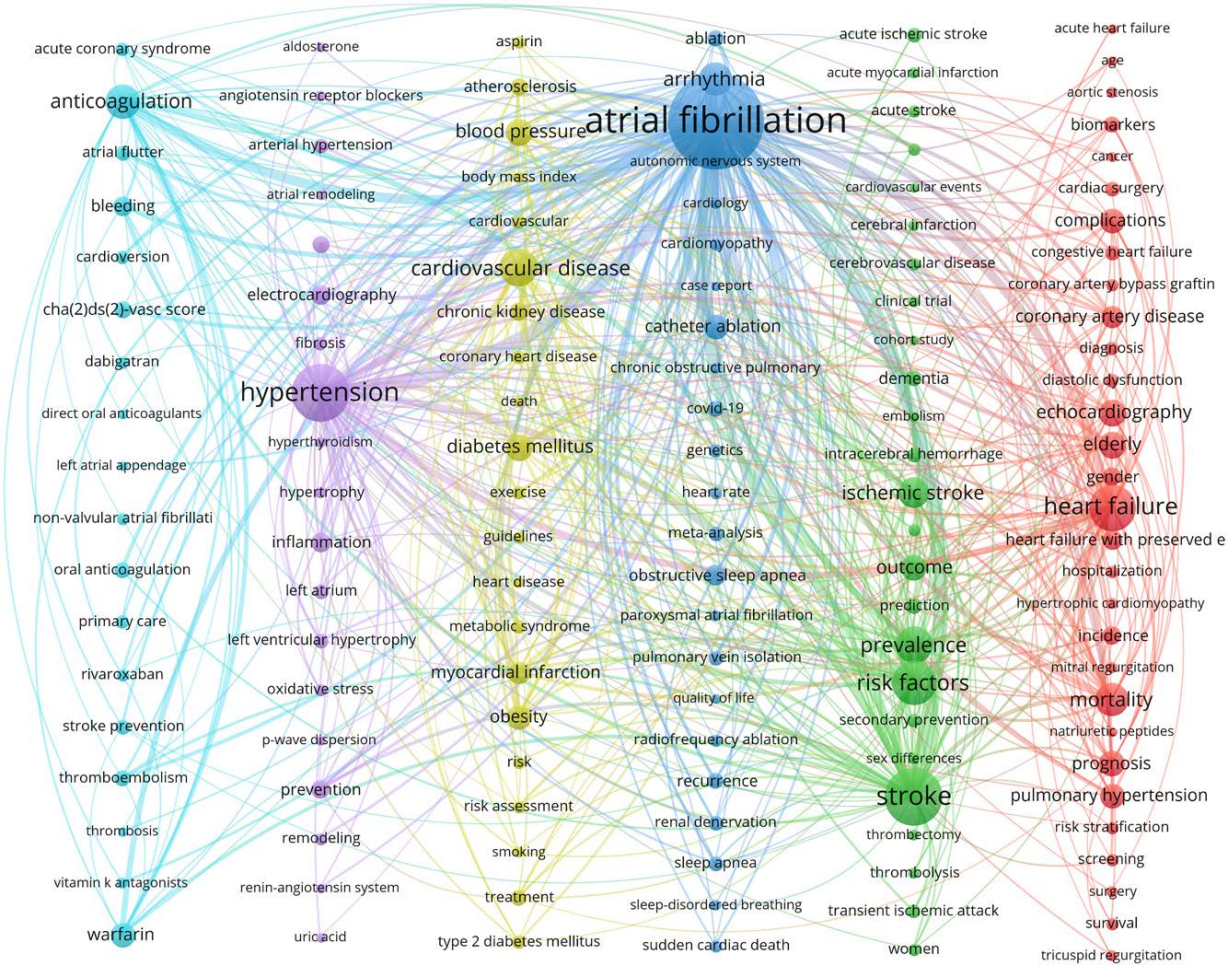
High-frequency keyword co-occurrence map.

To conduct a co-occurrence clustering analysis of keywords in the articles, we utilized VOSviewer software. The minimum occurrence threshold for each keyword was set at 25 times. atrial fibrillationter deduplication and merging, a total of 8773 keywords were obtained. From these, 136 keywords were selected to create a visualization map. In Figure 11, each node consists of a circle and a label. The size of the circle represents the frequency of the keyword, while the thickness of the connections between circles indicates the strength of the relationship between keywords. Nodes of different colors belong to different clusters, which represent distinct research directions.

Keywords are a high-level summary and refinement of the core themes and main contents of the research, and by analyzing the high-frequency keywords in the co-occurrence network, the main research structure and hot directions of the field can be quickly judged. Through the keyword co-occurrence clustering network analysis (Fig. 11), we can know the main research directions of hypertension combined with atrial fibrillation, that is, the research hotspots include the following 6 aspects: The red cluster is the research direction of heart failure caused by hypertension combined with atrial fibrillation and its echocardiography evaluation and treatment,^[32,33]^ where the high-frequency words include: heart failure, mortality, echocardiography, elderly, prognosis, pulmonary hypertension, complications, coronary artery disease, heart failure with preserved ejection fraction, incidence; The green cluster is the research direction of the mutual influence, prevention and treatment strategies of hypertension combined with atrial fibrillation and stroke,^[34,35]^ where the high-frequency words include: stroke, risk factors, prevalence, ischemic stroke, outcome, dementia, prediction, acute ischemic stroke, intracerebral hemorrhage, transient ischemic attack; The blue cluster is the research direction of diagnosis, treatment and prognosis evaluation of patients with hypertension combined with atrial fibrillation,^[36,37]^ where the high-frequency words include: atrial fibrillation, arrhythmia, catheter ablation, obstructive sleep apnea, ablation, recurrence, COVID-19, meta-analysis, sleep apnea, renal denervation; The yellow cluster is the research direction of risk factors, ablation treatment and exercise intervention of hypertension combined with atrial fibrillation,^[38,39]^ where the high-frequency words include: cardiovascular disease, diabetes mellitus, blood pressure, obesity, myocardial infarction, chronic kidney disease, atherosclerosis, treatment, metabolic syndrome, exercise; The purple cluster is the research direction of the relationship between hypertension-induced cardiac remodeling and atrial fibrillation occurrence and its intervention measures,^[40,41]^ where the high-frequency words include: hypertension, electrocardiography, inflammation, prevention, cardiovascular risk factors, left ventricular hypertrophy, arterial hypertension, left atrium, fibrosis, oxidative stress; The sky blue cluster is the research direction of anticoagulation treatment selection and risk assessment of patients with hypertension combined with atrial fibrillation,^[42,43]^ where the high-frequency words include: anticoagulation, warfarin, bleeding, cha(2)ds(2)-vasc score, oral anticoagulation, thromboembolism, atrial flutter, stroke prevention, acute coronary syndrome, cardioversion.

### 3.8. Keyword emergence analysis

**Figure 12.**
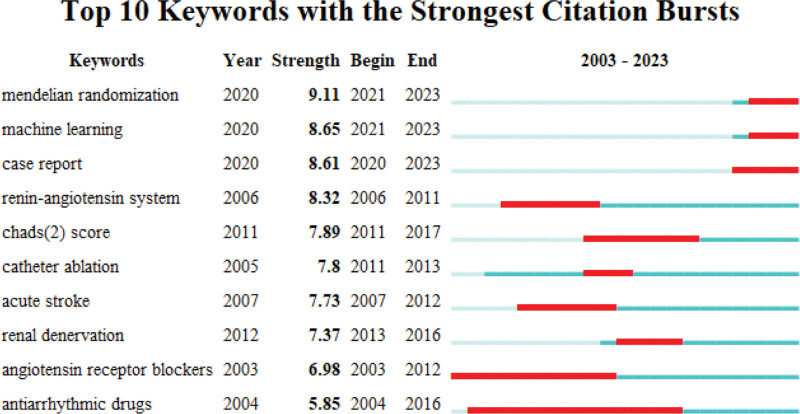
Keyword emergence analysis map.

Keyword emergence refers to the sudden surge in the occurrence frequency of a specific term over a defined period, serving as an indicator of cutting-edge areas within a research discipline. By calculating the growth rate of these keywords, we can discern the focal points and developmental trajectories of the field, ultimately offering fresh themes and novel concepts for future investigations. In Figure 12, the highlighted region represents the time interval of significant keyword emergence. The graph clearly portrays the accelerated advancement of the hypertension and atrial fibrillation domain, as evidenced by the emergence of keywords like Mendelian randomization, machine learning, and case reports within the past 3 years. This definite upswing indicates a noteworthy upsurge in scholarly articles pertaining to these terms, firmly establishing them as the trending areas of investigation in this particular field.

### 3.9. Keyword time zone analysis

**Figure 13.**
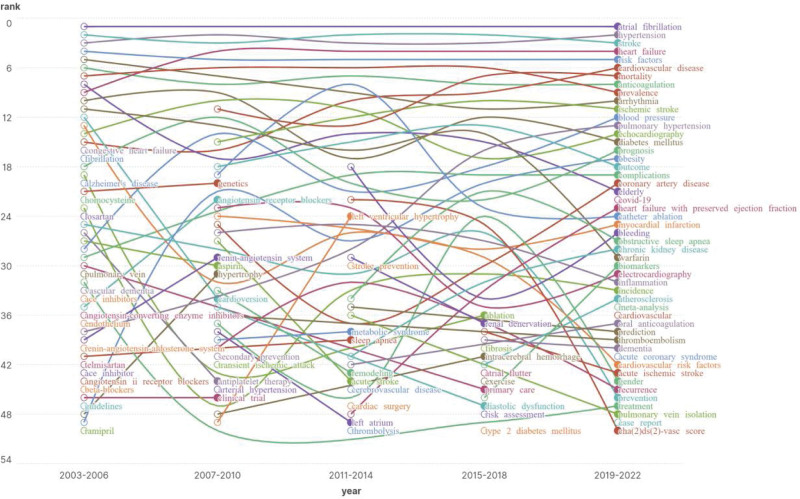
Keyword time zone map.

The analysis of keyword evolution involved dividing the keyword co-occurrence map into 4-year time periods. Within each time period, we identified the top 50 keywords based on frequency and created Figure 13, which visually represents the keyword time zone map. Time marks on the map are located at the bottom, indicating the progression of time from left to right. The curve fluctuation reflects changes in keyword rankings. Observing Figure 13, it becomes apparent that the period from 2003 to 2006 marked the initial phase of research in this field. Key focus areas during this time included atrial fibrillation, stroke, hypertension, risk factors, arrhythmia, anticoagulation, prevalence, elderly, and heart failure. These keywords indicate that research during this phase primarily centered around the epidemiology and associated mechanisms of hypertension and atrial fibrillation, as well as their roles as risk factors for heart failure and other cardiovascular diseases. Moving forward to the period from 2015 to 2018, we observe a shift in the main keywords toward biomarkers, acute ischemic stroke, dementia, and atherosclerosis. This shift in keyword prominence continues from 2019 to 2022, which suggests a gradual change in research focus toward exploring complications and prognosis in patients with hypertension and atrial fibrillation. Consequently, recent research in this field aims to establish and enhance risk assessment and management models for patients with hypertension and atrial fibrillation. Additionally, there is a growing emphasis on improving quality of life and preventive measures for these patients, along with an in-depth exploration of influencing factors and mechanisms related to associated risks.

## 4. Discussion

### 4.1. General information

This study analyzed a total of 7936 articles and found that there has been a consistent increase in the number of publications on hypertension and atrial fibrillation. The research in this field is becoming more comprehensive and receiving more attention. More than half of the studies are related to the cardiovascular system and cover various interdisciplinary fields. This diversity creates opportunities for interdisciplinary investigations.

The analysis of keyword prominence is a useful tool for identifying the main themes and current focuses of research. It helps summarize the overall landscape of studies and predict future research directions. The analysis shows that the research focus has changed over the years. From 2015 to 2018, the main areas of research were biomarkers, acute ischemic stroke, dementia, and atherosclerosis. In the past 3 years, the prominent keywords have been Mendelian randomization, machine learning, and case reports. This suggests that current research in this field includes case reports related to hypertension and/or atrial fibrillation. These reports are important in clinical research and practice as they help identify and discover new diseases or adverse reactions, understand disease mechanisms and treatments, provide references for disease management, contribute to medical education and training, and summarize cutting-edge therapeutic measures.^[44–46]^

Conducting Mendelian randomization studies to investigate the association between hypertension and atrial fibrillation has been a common approach. This method allows for exploring the impact of factors like elevated blood pressure, genetic susceptibility, and drug treatment on the risk of atrial fibrillation.^[47–49]^ A Mendelian randomization analysis conducted using data from the UK Biobank revealed a linear gradient,^[50]^ showing that higher systolic blood pressure increases the risk of atrial fibrillation by 19%. This effect is particularly pronounced in populations with high genetic susceptibility. Multiple genome-wide association studies datasets used in Mendelian randomization analysis have identified new gene variations that are associated with atrial fibrillation, some of which are also correlated with blood pressure levels.^[51]^ In a study by Liao et al,^[52]^ hypertension was found to be a risk factor for atrial fibrillation, indicating an increased risk of atrial fibrillation with elevated blood pressure. Furthermore, the study showed that systolic blood pressure is a risk factor for atrial fibrillation, while there is no apparent causal relationship between diastolic blood pressure and atrial fibrillation.

In the field of hypertension and atrial fibrillation research, machine learning has undergone rapid development. Artificial intelligence (AI)-based systems have been widely applied in cardiovascular imaging and cardiovascular disease risk prediction, leading to significant advancements in various aspects of the medical domain.^[53–55]^ Ebrahimzadeh et al conducted a small-scale study aiming to predict atrial fibrillation by analyzing heart rate variability in 53 patients’ electrocardiogram records using machine learning techniques.^[56]^ Another bioinformatics-based study utilized the Gene Expression Omnibus database for microarray meta-analysis to identify differentially expressed genes.^[57]^ The study introduced a correlation-based feature selection method based on machine learning to identify atrial fibrillation-related genes, successfully identifying 30 consistent trending biomarkers. With the proliferation of big data, algorithmic improvements, and the development of wearable technology, AI-guided clinical research is poised to become a turning point in the new era.

## 5. Strength and limitations

This study utilized bibliometrics and visualization analysis to examine the literature on hypertension combined with atrial fibrillation, providing insights into the research focus and trends in this field. However, there are certain limitations to this study: Only literature indexed in the Web of Science Core Collection database was included, potentially resulting in the omission of some relevant literature. The software analysis process lacked standardized setting procedures for time zone, threshold, and trimming methods. We attempted to approach accurate results through repeated exploration, but this may have atrial fibrillation affected the accuracy. In future research, we aim to introduce innovative methodologies, extract information from multiple databases, and employ different tools for analysis and comparison.

## 6. Conclusion

This study provides a comprehensive analysis of research on hypertension and atrial fibrillation from 2003 to 2022, revealing the developmental trends and future directions of the field. Through the analysis of 7936 related publications, we have identified a continuous growth in research on hypertension and atrial fibrillation, as well as key characteristics such as geographic distribution, core research institutions, and research focus. Currently, the research emphasis on hypertension combined with atrial fibrillation mainly centers on exploring pathophysiological mechanisms, optimizing treatment strategies for complications, and in-depth investigation of risk factors. Looking ahead, the research frontier is likely to shift toward exploring genetic and molecular mechanisms, deepening the understanding of the association between atrial fibrillation and related complications such as stroke, further revealing the role of oxidative stress and inflammatory responses in the progression of the disease, and utilizing Mendelian randomization studies to reveal causal relationships. Additionally, integrating artificial intelligence and genetics research will promote personalized treatment for hypertension and atrial fibrillation, moving toward the development of precision medicine.

## Acknowledgments

We are very grateful to all the people who participated in this study.

## Author contributions

**Data curation:** Nan Tang, Qiang Zhou, Shuang Liu, Kangming Li, Zhen Liu, Qingdui Zhang, Huamei Sun, Cheng Peng, Ji Hao.

**Formal analysis:** Nan Tang.

**Funding acquisition:** Huamei Sun, Ji Hao, Chunmei Qi.

**Investigation:** Qiang Zhou, Shuang Liu, Kangming Li, Zhen Liu, Qingdui Zhang, Huamei Sun, Cheng Peng, Ji Hao.

**Methodology:** Nan Tang, Qiang Zhou, Shuang Liu, Kangming Li, Zhen Liu, Qingdui Zhang, Huamei Sun, Cheng Peng, Ji Hao.

**Project administration:** Chunmei Qi.

**Software:** Nan Tang.

**Visualization:** Nan Tang.

**Writing – original draft:** Nan Tang.

**Writing – review & editing:** Ji Hao, Chunmei Qi.
